# Cellular Redox Homeostasis

**DOI:** 10.3390/antiox10091377

**Published:** 2021-08-28

**Authors:** Kristell Le Gal, Edward E. Schmidt, Volkan I. Sayin

**Affiliations:** 1 Institute of Clinical Sciences, Department of Surgery, Sahlgrenska Center for Cancer Research, University of Gothenburg, 405 30 Gothenburg, Sweden; kristell.le.gal.beneroso@gu.se; 2 Wallenberg Centre for Molecular and Translational Medicine, University of Gothenburg, 405 30 Gothenburg, Sweden; 3 Microbiology & Cell Biology, Montana State University, Bozeman, MT 59718, USA; eschmidt@montana.edu; 4 McLaughlin Research Institute, Great Falls, MT 59405, USA; 5 Laboratory of Redox Biology, University of Veterinary Medicine, 1078 Budapest, Hungary

**Keywords:** redox, homeostasis, oxidation, reduction, antioxidants, ROS, glutathione, thioredoxin

## Abstract

Cellular redox homeostasis is an essential and dynamic process that ensures the balance between reducing and oxidizing reactions within cells and regulates a plethora of biological responses and events. The study of these biochemical reactions has proven difficult over time, but recent technical and methodological developments have contributed to the rapid growth of the redox field and to our understanding of its importance in biology. The aim of this short review is to give the reader an overall understanding of redox regulation in the areas of cellular signaling, development, and disease, as well as to introduce some recent discoveries in those fields.

## 1. Introduction

Since the evolutionary appearance of photosynthesis led to the accumulation of oxygen in the atmosphere, life has needed to adapt to an environment wherein exposure to oxidative reactions happens on a regular basis. Consequently, new molecular mechanisms have been developed to regulate and maintain the balance between reducing and oxidizing reactions. This so-called redox homeostasis not only allowed life to continue in this new oxidizing environment but also triggered an explosion of biodiversity [1,2].

Contrary to what the name might suggest, redox homeostasis is a very dynamic process wherein the steadiness of the redox status within cells is maintained not by having a constant metabolism but rather by having a highly responsive system that senses changes in redox status and realigns metabolic activities to restore redox balance. Cellular redox biology has proven challenging to study, and the interactions between electron donors and acceptors are far more complex and difficult to map than once thought. The systems in charge of keeping this cellular redox balance, such as the glutathione, thioredoxin, NADPH-regenerating systems, and their associated enzymes, have been extensively studied and characterized. The reader is kindly referred to references [3,4,5] for extensive reviews on this topic.

**Figure 1:**
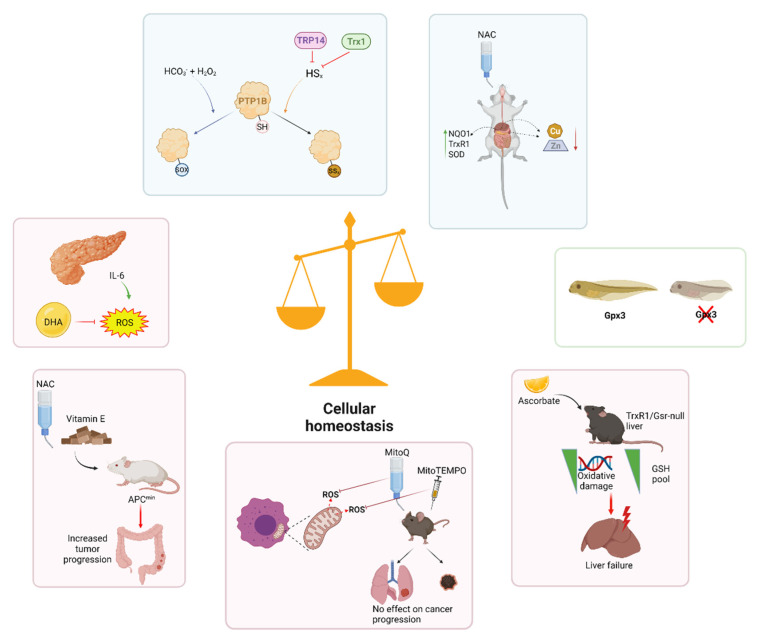
Redox homeostasis plays key roles in the regulation of cell signaling, development, health, and disease. Examples of recent research on cellular redox homeostasis in the areas of cellular signaling, development, and pathology. Created with BioRender.com. TRP14: Trx-like protein of 14 kDa; Trx1: thioredoxin-1; PTP1B: protein tyrosine phosphatase-1B; SH: thiol; SOX: SRG-related high mobility group-box transcription factors; NAC: *N*-acetyl cysteine; NQO1: NADPH-quinone oxidase; SOD: superoxidase dismutase; IL-6: interleukin-6; ROS: reactive oxygen species; DHA: dehydroascorbate; Gpx3: glutathione peroxidase 3; APC: adenomatous polyposis coli protein; TrxR1: thioredoxin reductase; Gsr: glutathione reductase; GSH: reduced glutathione.

Redox interactions are responsible for the regulation of diverse biological processes, including metabolism, cell death, differentiation and development, immune responses, circadian rhythm, and others. This short review on cellular redox homeostasis provides an overview of some of the most recent advances in our understanding of how redox homeostasis is maintained, and what roles redox modulation plays in cellular signaling, development, and pathology (Figure 1).

## 2. Cellular Signaling

Reactive oxygen species (ROS) are strong oxidants that include molecular oxygen (O_2_) and the sequential series of 1-electron reduction steps of O_2_ leading to species more oxidized than water, in order: superoxide radical (^●^O^2−^), hydrogen peroxide (H_2_O_2_), and hydroxyl radical (HO^●^). These are generated in aerobic organisms primarily as a collateral result of utilizing oxygen as the final electron acceptor in the electron transport chain (ETC) to produce energy [6] or as a result of other metabolic processes, including glycolysis or β-oxidation of fatty acids. However, in other cases, ROS can be deliberately generated by the regulated activities of NADPH oxidoreductases [7]. ROS can act as secondary messengers by, for example, reversibly oxidizing cysteine residues in proteins, resulting in their activation or inactivation [8]. They can also alter DNA and often transit cellular membranes via aquaporins or other channels [9,10,11].

ROS are well-known regulators of signaling cascades by, for instance, inhibiting phosphatases or inhibiting antioxidant proteins normally bound to kinases [12]. Highly reactive cysteine residues in the active sites of protein tyrosine phosphatases (PTPs) are sensitive to oxidative inhibition by hydrogen peroxide (H_2_O_2_). Moreover, recent studies have shown that some protein-cysteine residues might be even more susceptible to inactivation by persulfides or polysulfides as compared to by H_2_O_2_, resulting in a cysteine-persulfide (Cys–SSH) or cysteine-polysulfide (Cys–S_n_SH), and that this is a relatively abundant modification of native proteins in cell cultures and in mouse liver [13]. Reaction of a cysteine-thiol (-SH) with H_2_O_2_ forms an unstable sulfenic acid (–SOH) residue that usually will spontaneously react with another vicinal thiol to form a disulfide (-S-S-). Per/poly-persulfidation of a cysteine-thiol also forms a disulfide bond (Cys–S–SH). In either case, the disulfide bond is readily reversible by disulfide-reductase enzymes of the thioredoxin (Trx) or glutaredoxin (Grx) families [13]. In the presence of higher concentrations of H_2_O_2_, a cysteine-thiol can overoxidize to sulfinic- (–SO_2_H) or sulfonic (–SO_3_H)-acid species, which cannot be reduced by disulfide reductases [14]. However, if a cysteine is first modified by per/polysufidation, subsequent overoxidation by high concentrations of H_2_O_2_ will result in the formation of a cysteine-per/polysufinic (Cys–S_n_SO_2_H) or -sulfonic acid (Cys–S_n_SO_3_H), which each, in addition to the terminal overoxidized sulfur, retain a disulfide linkage involving the proximal cysteine sulfur residue. Importantly, this disulfide linkage remains a good substrate for Trx and Grx disulfide reductases [13], allowing this type of overoxidation to be repaired. Indeed, it has been suggested that cells might retain a pool of some critical enzymes with their active site cysteine residue in a per/polysulfidated state specifically to facilitate recovery from oxidative insults [13]. In a very recent advance to our understanding of redox regulation within cells, a study presented by Dagnell et al. shows that regulatory inhibition of the PTP active site cysteine by either H_2_O_2_ oxidation or per/polysulfidation might require bicarbonate as a cofactor [15].

In order to maintain redox homeostasis in changing conditions, cells possess stress-response systems that are sensitive to cytosolic levels of either ROS or electrophilic toxins. Many of the downstream effector genes in these systems contain a sequence known as the antioxidant response element (ARE), which is recognized by the transcription factor Nrf2, the master regulator of the endogenous antioxidant response. Nrf2, in turn, is post-translationally regulated by the cytosolic Nrf2-interacting protein Keap1, which determines whether Nrf2 will be directed to the proteasome by the E3 ubiquitin ligase Cul3 and degraded or be allowed to transit to the nucleus where it will heterodimerize with small Maf proteins and activate ARE-containing genes [16,17]. In addition to regulating the expression of antioxidant enzymes, Nrf2 controls the expression of enzymes more peripherally associated with maintaining redox homeostasis. This includes downregulating anabolic enzymes that would compete with the redox systems for NADPH, upregulating exporters that might help eliminate electrophilic toxins from the cell, and modulating enzymes involved in controlling heme metabolism and iron homeostasis [18]. In addition to iron, other trace metals such as selenium (Se), copper (Cu), and zinc (Zn) are essential to the function of antioxidant enzymes [19]. Recently, it has been shown that Zn-dependent modulation of Nrf2 regulates the cellular levels of some of these trace metals. Interestingly, in cell culture models, N-acetylcysteine (NAC), a commonly prescribed antioxidant with metal-chelating properties, inhibited Zn-induced activation of Nrf2 by depleting cellular pools of Zn and Cu. However, in mouse models, the effects of NAC administration showed tissue-specific effects, with Cu, Zn, and Nrf2 target gene activity decreasing in the liver and spleen, yet conversely increasing in the duodenum [20].

## 3. Development

To understand the impact of redox regulation during development, special attention needs to be paid to redox spatiotemporal interactions. For instance, in plants, the fine interplay between phytohormones, redox signaling, and cell metabolism enables the dynamic regulation of cell growth and division [21]. Differences in cytosolic and nuclear ROS levels control apical root growth through the renewal and differentiation of stem cells [22]. ROS also act as a positive signal to promote root hair growth and control the germination of seeds [23]. In contrast, primary root development is regulated majorly by reduced glutathione (GSH) [24]. Both the GSH and the thioredoxin system control bud dormancy, burst, and flowering [25].

In mammals, there is a change from ATP production by oxidative phosphorylation to ATP production by glycolysis during the transition to blastocyst stage, which might reflect a shift to a more reduced state [26]. Additionally, decreased levels of GSH appear to increase during oocyte maturation and are also associated with favorable outcomes in fertilization and in vitro embryonic development [27]. In contrast, constant activation of the antioxidant transcription factor Nrf2 in mice is postnatally lethal [28]. However, antioxidant supplementation has been shown to rescue newborn lethality in mice presenting lung maturation defects and respiratory insufficiency [29]. Adding to our understanding of the roles of redox modulation in development, Lee and colleagues [30] have shown the importance of the antioxidant enzyme glutathione peroxidase-3 (GPx3) in posterior vertebrate embryogenesis in frogs, expanding the pool of data demonstrating the implication of redox processes in segmentation and organogenesis.

## 4. Disease

The involvement of ROS and reactive nitrogen species (RNS) in various human diseases is well established, yet their exact contribution to the development of the different pathogenies is far from elucidated. In fact, the literature often presents them as a double-edged sword with both beneficial and deleterious effects [4]. For instance, the production of ROS during inflammatory responses plays a critical role in microbial defense, yet excessive or inappropriately regulated ROS production can also damage tissue. Indeed, sustained ROS production has been reported in a number of inflammatory diseases, with consequent exhaustion of antioxidant systems [31,32]. Clinical interventions with antioxidant supplementation to compensate for excessive ROS production in renal disease have proven more difficult than expected, with highly variable results depending on the compound used and stage of the disease [33]. Recently, promising preclinical results have been reported by using dehydroascorbate (DHA) as a modulator of the Nrf2 response to dampen oxidative damage in a model of acute pancreatitis [34], but these findings are yet to be translated to clinical settings. Interestingly, another report (29) highlights the caveat of antioxidant treatments sometimes exacerbating oxidative stress-induced pathologies. In this study, the researchers used a mouse model of acute spontaneous liver failure associated with hepatic reductase system deficiency to show that ascorbate supplementation, despite diminishing DNA damage and oxidative stress markers, also dramatically depleted hepatic GSH levels and significantly increased the probability of acute liver failure [35]. This counterintuitive notion of antioxidants worsening oxidative stress-related pathologies has been similarly shown in the context of arthritis, wherein a gene polymorphism associated with a reduced oxidative burst response was linked to an autoimmune mechanism [36].

ROS-damaging effects are often associated with age-related disorders, such as neurodegenerative disorders, and cancer [4]. Even there, however, and perhaps more notably in the latter, clinical interventions aiming to compensate for excessive oxidative damage by dietary supplementation of antioxidants have had mixed effects [37,38,39,40,41,42]. To further complicate the picture, there is a lack of consensus in preclinical data. However, an increasing number of studies show that antioxidants can benefit the progression of cancer in endogenous models of cancer [43,44,45,46,47,48]. In fact, loss-of-function mutations in Keap1 resulting in constitutive Nrf2 activation have been observed in a substantial portion of lung cancer patients [49,50] and validated as driver mutations in mouse models of cancer [51,52], showing that tumor cells find ways to hijack the endogenous antioxidant systems. In a study conducted by Zou and colleagues, a mouse model of familial colorectal cancer was used in conjunction with dietary supplementation of either NAC or vitamin E, which revealed that antioxidant supplementation in Apc^Min/+^ mice enhanced parameters of intestinal tumor progression without affecting tumor initiation [47].

One plausible reason for the disappointing effects of dietary interventions with antioxidants could be that the antioxidants used were not reaching one of the major sites of ROS production in the cells, the mitochondria [53]. Hence, new strategies aiming to design redox compounds that would target the mitochondria have emerged [54]. Interestingly, these mitochondria-targeted compounds have shown promising results in some areas of disease, such as cardiovascular and neurodegenerative disorders [55,56,57,58,59]. Nevertheless, some studies show that, in different preclinical tumor models, these drugs can have diverse and often unexpected impacts on cancers [60,61,62,63]. For instance, a recent report using MitoQ and MitoTEMPO, two independent mitochondria-targeted antioxidants in endogenous mouse models of malignant melanoma and lung cancer, as well as in a panel of human cancer cell lines, showed no signs of antitumorigenic effects [60].

## 5. Conclusions

It is our hope that this short review on cellular redox homeostasis by presenting some of the latest advances on the topic, will give the reader an up-to-date overview of the field and highlight both the complexity and the importance of redox regulation in health and disease.
